# Effect of iron supplementation on brain myelination and neurodevelopmental outcomes in children aged 0–3 years: a systematic review protocol

**DOI:** 10.3389/fped.2026.1891295

**Published:** 2026-08-25

**Authors:** Anju Sinha, Yamini Priyanka Kuruba, Pradeep Kumar, Rounik Thalukdar, Subodh Gupta, Alok Pradhan, Kushagra Shiromani, Jeeva Shankar

**Affiliations:** 1 Division of Reproductive, Child Health and Nutrition, Indian Council of Medical Research, New Delhi, India; 2 Department of Pediatrics, India ICMR- National Institute of Child Health and Development Research, New Delhi, India; 3 Clinical Research Unit, All India Institute of Medical Sciences, New Delhi, India; 4 Department of Community Medicine, Mahatma Gandhi Institute of Medical Sciences, Sewagram, India; 5 Department of Pediatrics, Independent Consultant, Pediatrics, New Delhi, India

**Keywords:** brain myelination, children, infants, iron deficiency, iron supplementation, neurodevelopment, protocol, systematic review

## Abstract

**Background:**

Iron deficiency is one of the most prevalent micronutrient deficiencies globally, affecting nearly 40% of children aged 6–59 months. Iron plays an essential role in early brain development, particularly in myelination, neurotransmitter synthesis and energy metabolism. Deficiency during critical developmental periods may impair cognitive, motor and behavioral outcomes. Although iron supplementation improves hematological parameters, its effects on brain myelination and neurodevelopmental outcomes in children aged 0–3 years remain unclear.

**Objective:**

To evaluate the effects of iron supplementation administered during pregnancy or early childhood (0–3 years) on brain myelination and neurodevelopmental outcomes in children.

**Methods:**

This systematic review protocol follows PRISMA 2020 and Cochrane Handbook recommendations and has been prospectively designed for registration in PROSPERO. We will search PubMed, EMbase, Scopus, CENTRAL, PsycINFO, CINAHL and Web of Science for randomized controlled trials, quasi-experimental studies, cohort studies, and observational studies evaluating iron supplementation in pregnant women or children aged 0–3 years. Interventions will include iron supplementation in any form and dosage, compared with placebo, standard care, or alternative interventions. Primary outcomes include cognitive, motor, language, executive function, memory and myelination outcomes assessed through validated neurodevelopmental tools and neuroimaging modalities. Secondary outcomes include adverse events. Risk of bias will be assessed using ROB 2 and ROBINS-I tools and certainty of evidence will be evaluated using the GRADE approach.

**Discussion:**

This review will synthesize current evidence regarding the role of iron supplementation in brain myelination and neurodevelopment during early childhood. The findings may inform clinical practice and public health policy, particularly in low- and middle-income settings where iron deficiency is highly prevalent.

**Clinical Trial Registration:**

https://www.crd.york.ac.uk/PROSPERO/view/CRD420261388359, Registration ID: CRD420261388359.

## Introduction

Iron deficiency is among the most common micronutrient deficiencies globally, particularly affecting pregnant women and young children. According to the World Health Organization, approximately 40% of children aged 6–59 months suffer from iron deficiency (1, 2). Iron plays a vital role in several physiological and neurological processes, including oxidative metabolism, neurotransmitter synthesis, DNA synthesis, cellular respiration, and myelination (3, 4).

The first 1,000 days of life represent a critical window for brain development. During this period, rapid neurodevelopment occurs, including axonal growth, synaptogenesis and myelination (5, 6). Iron deficiency during fetal and early postnatal life has been associated with impaired neurodevelopment, including deficits in cognition, memory, motor skills and socio-emotional development (7, 8).

Iron is an essential cofactor for oligodendrocyte function and myelin synthesis (9, 10). Experimental studies have demonstrated that iron deficiency may disrupt myelination and neural transmission (11). Although previous systematic reviews have examined the effects of iron supplementation on psychomotor and cognitive outcomes, evidence specifically related to brain myelination remains limited (12, 13).

Recent advances in neuroimaging, including diffusion tensor imaging (DTI), magnetization transfer imaging (MTI), and myelin water imaging (MWI), provide opportunities to evaluate structural brain changes associated with iron status and supplementation (14, 15). However, evidence integrating these outcomes remains sparse.

Therefore, this systematic review aims to synthesize current evidence on the effects of iron supplementation during pregnancy and early childhood on brain myelination and neurodevelopmental outcomes in children aged 0–3 years (16, 17).

## Methods

### Protocol and registration

This systematic review protocol has been developed in accordance with the Preferred Reporting Items for Systematic Reviews and Meta-Analyses (PRISMA) 2020 statement (18) and the methodological recommendations outlined in the Cochrane Handbook for Systematic Reviews of Interventions (19, 20). The review methodology has been prospectively designed to ensure transparency, reproducibility and methodological rigor (21). The protocol is registered in the International Prospective Register of Systematic Reviews (PROSPERO) with registration ID: PROSPERO 2026 CRD420261388359.

### Eligibility criteria

#### Types of studies

This review will include randomized controlled trials (RCTs), cluster-randomized controlled trials and cross-over randomized trials, with data from only the first phase of cross-over studies being considered to minimize carry-over effects (22). In addition, quasi-randomized studies, quasi-experimental studies, prospective cohort studies and relevant observational studies will be included because of the anticipated limited availability of high-quality randomized evidence in this research area (23, 24). Grey literature, conference abstracts, dissertations, and unpublished studies will also be considered if sufficient methodological and outcome-related details are available to determine eligibility and assess study quality.

#### Types of participants

Eligible participants will include pregnant women during antenatal or perinatal periods, neonates, preterm infants, infants and children aged from birth up to 3 years. Both male and female participants from hospital, community or primary healthcare settings will be considered eligible. Studies involving participants with significant congenital neurological abnormalities, severe chronic illnesses unrelated to iron deficiency or conditions known to independently affect neurodevelopment will be excluded unless outcome data are reported separately for eligible participants (25).

#### Types of interventions

The intervention of interest will be iron supplementation administered in any form, including tablets, capsules, syrups, drops, or fortified formulations (26). Iron supplementation may be provided either during pregnancy to mothers or directly to neonates, infants, and young children aged 0–3 years. Studies evaluating different doses, durations, formulations, or timing of iron supplementation will also be considered eligible. Because brain myelination progresses rapidly during fetal life and the first 1,000 days after birth, the timing of iron supplementation may substantially influence neurodevelopmental outcomes. Iron administered during pregnancy, the neonatal period, infancy, or early childhood may produce different biological effects owing to developmental differences in oligodendrocyte maturation, myelin formation, and neuronal connectivity. Similarly, the duration of supplementation may influence treatment effectiveness, as prolonged supplementation may result in greater replenishment of iron stores and sustained support of neurodevelopment. Therefore, studies will be categorized according to both the timing and duration of the intervention whenever sufficient data are available for analysis.

### Comparator

Eligible comparator groups will include placebo, standard care, no intervention, vitamin C supplementation, alternative nutritional interventions, or different iron supplementation regimens. Trials comparing varying doses, frequencies, or durations of iron supplementation will also be included where relevant (27).

### Outcomes

#### Primary outcomes

The primary outcomes of interest will include neurodevelopmental and neurophysiological outcomes associated with early brain development. These will include cognitive development, motor development, language development, executive functioning, memory and brain myelination (28, 29).

Brain myelination and neurophysiological maturation will be assessed using validated modalities such as auditory brainstem response (ABR), visual evoked potential (VEP) (30, 31), and advanced neuroimaging techniques including myelin water imaging (MWI), diffusion tensor imaging (DTI), and magnetization transfer imaging (MTI) (14, 15, 32). Standardized neurodevelopmental assessment tools such as the Bayley Scales of Infant and Toddler Development (33) and other validated developmental scales will also be considered (34, 35). The conceptual definitions, constructs, measurement scales, and relevant subscales for all critical neurodevelopmental outcomes included in this review are summarized in Table 1.

**Table 1:** Detailed electronic search strategies used across bibliographic databases and grey literature sources for identification of studies evaluating the effects of iron supplementation on brain myelination and neurodevelopmental outcomes in children aged 0–3 years.

Database	Search Strategy
PubMed/MEDLINE	((“Iron”[Mesh] OR “Iron Compounds”[Mesh] OR iron[tiab] OR ferric[tiab] OR ferrous[tiab]) AND (“Dietary Supplements”[Mesh] OR supplementation[tiab] OR supplement*[tiab] OR fortification[tiab]) AND (“Brain”[Mesh] OR “Myelin Sheath”[Mesh] OR myelin*[tiab] OR myelination[tiab] OR neurodevelopment*[tiab] OR cognition[tiab] OR cognitive[tiab] OR language[tiab] OR motor[tiab] OR memory[tiab]) AND (“Pregnant Women”[Mesh] OR pregnancy[tiab] OR antenatal[tiab] OR prenatal[tiab] OR maternal[tiab] OR infant*[tiab] OR neonat*[tiab] OR toddler*[tiab] OR child*[tiab]))
EMbase	(‘iron'/exp OR iron:ti,ab OR ferric:ti,ab OR ferrous:ti,ab) AND (‘dietary supplement'/exp OR supplementation:ti,ab OR supplement*:ti,ab OR fortification:ti,ab) AND (‘brain development'/exp OR ‘myelin sheath'/exp OR myelin*:ti,ab OR myelination:ti,ab OR neurodevelopment*:ti,ab OR cognition:ti,ab OR language:ti,ab OR motor:ti,ab OR memory:ti,ab) AND (‘pregnant woman'/exp OR pregnancy:ti,ab OR infant*:ti,ab OR neonat*:ti,ab OR toddler*:ti,ab OR child*:ti,ab)
Cochrane CENTRAL	[(mh Iron) OR iron:ti,ab,kw OR ferric:ti,ab,kw OR ferrous:ti,ab,kw] AND [(mh “Dietary Supplements”) OR supplementation:ti,ab,kw OR supplement*:ti,ab,kw OR fortification:ti,ab,kw] AND [(mh “Myelin Sheath”) OR myelin*:ti,ab,kw OR neurodevelopment*:ti,ab,kw OR cognition:ti,ab,kw OR motor:ti,ab,kw OR language:ti,ab,kw] AND [(mh Pregnancy) OR pregnancy:ti,ab,kw OR infant*:ti,ab,kw OR child*:ti,ab,kw]
Scopus	TITLE-ABS-KEY ((iron OR ferric OR ferrous) AND (supplement* OR supplementation OR fortified OR fortification) AND (myelin* OR myelination OR neurodevelopment* OR cognition OR language OR motor OR memory OR “executive function”) AND (pregnancy OR antenatal OR prenatal OR maternal OR infant* OR neonat* OR toddler* OR child*))
PsycINFO	(DE “Iron” OR iron.ti,ab. OR ferric.ti,ab.) AND (DE “Dietary Supplements” OR supplement*.ti,ab. OR supplementation.ti,ab.) AND (DE “Neurodevelopment” OR myelin*.ti,ab. OR cognition.ti,ab. OR language.ti,ab. OR motor.ti,ab. OR memory.ti,ab.) AND (pregnancy.ti,ab. OR infant*.ti,ab. OR child*.ti,ab.)
CINAHL	(MH “Iron+” OR iron TI OR iron AB OR ferric TI OR ferric AB) AND (MH “Dietary Supplements+” OR supplement* TI OR supplement* AB) AND (MH “Child Development+” OR MH “Myelin Sheath+” OR myelin* TI OR neurodevelopment* TI OR cognition TI OR motor TI OR language TI) AND (MH “Pregnancy+” OR infant* TI OR neonat* TI OR child* TI)
Web of Science	TS = ((iron OR ferric OR ferrous) AND (supplement* OR supplementation OR fortification OR fortified) AND (myelin* OR myelination OR neurodevelopment* OR cognition OR language OR motor OR memory OR “executive function”) AND (pregnancy OR antenatal OR infant* OR neonat* OR child*))
ClinicalTrials.gov	Condition or disease: Neurodevelopment OR Brain Development OR Myelination; Other terms: Iron supplementation OR iron therapy OR iron fortified
WHO ICTRP	(iron supplementation OR iron fortified OR iron therapy) AND (neurodevelopment OR brain development OR myelination OR cognition) AND (infant OR child OR pregnancy)
ProQuest Dissertations & Theses	(iron supplementation OR iron fortified OR iron therapy) AND (neurodevelopment OR myelination OR cognitive development OR motor development) AND (infant* OR neonat* OR toddler* OR child* OR pregnancy)

#### Secondary outcomes

Secondary outcomes will include adverse events associated with iron supplementation, including gastrointestinal intolerance, constipation, nausea, vomiting, diarrhea and outcomes related to iron overload or toxicity where reported.

### Search strategy

A comprehensive and systematic search strategy will be implemented to identify all relevant studies Table 2. Electronic databases including PubMed/MEDLINE, EMbase, Cochrane CENTRAL, Scopus, PsycINFO, CINAHL and Web of Science will be searched. The search strategy will incorporate Medical Subject Headings (MeSH) terms, keywords and Boolean operators related to iron supplementation, neurodevelopment, brain myelination, infants and early childhood (36).

**Table 2:** Conceptual definitions, key constructs, and standardized measurement scales/subscales used for assessment of critical neurodevelopmental and neurophysiological outcomes in children aged 0–3 years included in the review. The table summarizes commonly used validated developmental, behavioral, neurophysiological, and neuroimaging assessment tools applied across studies evaluating the effects of iron supplementation on early brain development and myelination.

Domain	Conceptual Definition	Key Constructs	Measurement Scales/Subscales
Cognitive Development	Mental processes involved in acquiring knowledge, understanding, reasoning, learning, and information processing, aligned with NIH Toolbox and Cattell–Horn–Carroll (CHC) theory frameworks.	Verbal comprehension, visual–spatial processing, working memory, attention, processing speed, problem-solving, concept formation, object permanence, memory.	BSID: Cognitive Scale GMDS: Performance, Eye–Hand Coordination MSEL: Visual Reception ASQ: Problem Solving Additional Tools: Bayley Scales of Infant Development, Wechsler Preschool and Primary Scale of Intelligence, Mullen Scales of Early Learning
Motor Development	Development of gross and fine motor skills based on developmental surveillance frameworks such as the Bayley Scales and WHO Motor Milestones Checklist.	Gross motor: sitting, crawling, walking, reaching Fine motor: grasping, pincer grip, hand–eye coordination	BSID: Fine and Gross Motor GMDS: Locomotor, Eye–Hand Coordination MSEL: Fine and Gross Motor ASQ: Gross and Fine Motor Additional Tools: Alberta Infant Motor Scale (AIMS), Peabody Developmental Motor Scales (PDMS)
Neurophysiological Development	Functional and structural maturation of neural pathways and neurodevelopmental systems, including electrophysiological and neuroimaging-based maturation.	Brain myelination, neuroelectric activity, sensory processing, auditory and visual pathways, reflexes.	ABR: Auditory Brainstem Response VEP: Visual Evoked Potential EEG/ERP: Brain electrical activity and event-related potentials MRI-based Measures: Fractional anisotropy, myelin water fraction, diffusion tensor imaging
Language Development	Ability to understand, process, and express verbal and non-verbal communication.	Receptive language, expressive language, vocabulary acquisition, communication skills.	BSID: Receptive and Expressive Language GMDS/ABAS: Hearing and Speech MSEL: Receptive and Expressive Language ASQ: Communication Additional Tools: MacArthur–Bates Communicative Development Inventories (CDI), Preschool Language Scale (PLS), Bayley Language Index
Socioemotional Development	Development of emotional regulation, social interaction, attachment, and behavioral adaptation.	Emotional regulation, interpersonal relationships, social responsiveness, behavior regulation.	BITSEA: Brief Infant-Toddler Social and Emotional Assessment ASQ:SE: Ages and Stages Questionnaire–Social Emotional SDQ: Strengths and Difficulties Questionnaire CBCL: Behavioral and emotional problems
Executive Function	Higher-order cognitive processes involved in goal-directed behavior and self-regulation.	Working memory, inhibition, planning, cognitive flexibility, attention shifting.	NEPSY-II: Executive Function and Attention CBCL: Attention Problems
Memory	Processes involved in storage, retention, and retrieval of information.	Working memory, recognition memory, recall ability.	BSID/MSEL: Memory-related tasks Research-Based Tasks: Deferred imitation, paired comparison paradigms
Adaptive Behavior	Application of learned skills to everyday functioning and environmental interaction.	Self-care, communication, instruction following, social adaptation, safety awareness.	Vineland Adaptive Behavior Scales ABAS: Adaptive Behavior Assessment System

BSID, Bayley Scales of Infant and Toddler Development; GMDS, Griffiths Mental Developmental Scales; MSEL, Mullen Scales of Early Learning; ASQ, Ages and Stages Questionnaire; EEG, Electroencephalogram; ERP, Event-Related Potentials; ABR, Auditory Brainstem Response; VEP, Visual Evoked Potential; NEPSY-II, A Developmental Neuropsychological Assessment; CBCL, Child Behavior Checklist; BITSEA, Brief Infant-Toddler Social and Emotional Assessment; SDQ, Strengths and Difficulties Questionnaire; ABAS, Adaptive Behavior Assessment System.

To identify unpublished and ongoing studies, grey literature sources including ClinicalTrials.gov, the World Health Organization International Clinical Trials Registry Platform (WHO ICTRP), conference proceedings and ProQuest Dissertations and Theses will also be searched. Additionally, the reference lists of included studies and relevant systematic reviews will be manually screened to identify any additional eligible studies.

### Study selection

All retrieved records will be uploaded into Covidence software followed by removal of duplicates for screening and study management. Two independent reviewers will screen titles and abstracts for eligibility, followed by full-text assessment of potentially relevant studies. Any disagreements between reviewers will be resolved through discussion and consensus, with consultation from a third reviewer where necessary. The study selection process will be documented using a PRISMA 2020 flow diagram (18). This will also serve as the primary framework for documenting the study selection process, including the number of records identified, screened, assessed for eligibility, and included in the review, together with reasons for exclusion at the full-text screening stage.

### Data extraction

Data extraction will be independently conducted by two reviewers using a standardized and piloted data extraction form (20). Extracted information will include study characteristics, participant demographics, intervention details, comparator characteristics, outcome measures, duration of follow-up, and findings relevant to the review objectives. Information related to study methodology and risk of bias will also be collected. Any discrepancies in extracted data will be resolved through discussion and consensus.

### Risk of bias assessment

The methodological quality and risk of bias of included studies will be independently assessed by two reviewers. Randomized controlled trials will be evaluated using the Cochrane Risk of Bias 2 (ROB 2) tool (37), while non-randomized studies will be assessed using the ROBINS-I tool (38). Domains assessed will include bias arising from the randomization process, deviations from intended interventions, missing outcome data, outcome measurement, and selective reporting. Disagreements between reviewers will be resolved through discussion or consultation with a third reviewer.

### Data synthesis

Where studies are sufficiently homogeneous in terms of participants, interventions, and outcomes, quantitative synthesis will be performed using Review Manager Software (39). For continuous outcomes, mean differences (MD) or standardized mean differences (SMD) with 95% confidence intervals will be calculated. For dichotomous outcomes, risk ratios (RR), odds ratios (OR) and corresponding 95% confidence intervals will be reported (40, 41).

Random-effects meta-analysis models will be used where appropriate to account for between-study variability. Where meta-analysis is not feasible because of substantial heterogeneity or insufficient data, findings will be summarized narratively following the Synthesis Without Meta-analysis (SWiM) reporting guidelines (42, 43).

### Assessment of heterogeneity

Statistical heterogeneity among included studies will be assessed using the Chi-square test, I^2^ statistic, and Tau^2^ statistic (44). Heterogeneity will be interpreted according to standard Cochrane recommendations. Where sufficient data are available, subgroup analyses will be conducted based on timing of supplementation, dosage of iron supplementation, duration of intervention, study design, and geographic setting, including comparisons between high-income and low- and middle-income countries.

Where sufficient data are available, subgroup analyses will be performed according to the timing of iron supplementation (antenatal, neonatal, infancy, or early childhood), duration of intervention (≤3 months, >3–6 months, and >6 months, or as reported in the included studies), dosage, formulation, study design, participant characteristics (preterm versus term infants, iron-deficient versus non-iron-deficient populations), and geographical setting. Additional subgroup analyses will be undertaken for studies reporting brain myelination outcomes using advanced neuroimaging techniques, including DTI, MWI, MTI, MRI-based quantitative myelin measures, and other validated neuroimaging biomarkers. Where quantitative pooling is not appropriate because of differences in imaging protocols or outcome measures, findings will be synthesized narratively using the SWiM guidelines (42).

### Sensitivity analysis

Sensitivity analyses will be conducted to evaluate the robustness of the pooled findings. These analyses will include exclusion of studies with high risk of bias, exclusion of outlier studies, and exclusion of studies with missing or imputed data. The influence of study design and methodological quality on pooled effect estimates will also be explored (45, 46).

### Certainty of evidence

The certainty of evidence for each major outcome will be assessed using the Grading of Recommendations Assessment, Development and Evaluation (GRADE) approach (47, 48). Evidence will be categorized as high, moderate, low, or very low certainty based on assessment of risk of bias, inconsistency, indirectness, imprecision, and publication bias (49).

## Discussion

Iron deficiency during pregnancy and early childhood remains a major global public health challenge, particularly in low- and middle-income countries (1). Although iron supplementation is widely recommended to prevent anemia and improve child health outcomes, evidence specifically addressing its effects on brain myelination and neurodevelopment remains fragmented and inconsistent (12, 13). This systematic review aims to comprehensively synthesize available evidence regarding the impact of iron supplementation on cognitive, motor, language, executive, and neurophysiological development in children aged 0–3 years (50).

This review is expected to address important evidence gaps related to the optimal timing, dosage, formulation, and duration of iron supplementation during early life. Since myelination occurs most rapidly during fetal development and the first 1,000 days after birth, identifying the developmental period during which iron supplementation provides the greatest neurodevelopmental benefit may have important implications for clinical practice and public health nutrition programmes. The planned subgroup analyses according to intervention timing and duration are expected to provide clinically relevant evidence for optimizing supplementation strategies. Furthermore, studies employing advanced neuroimaging techniques such as diffusion tensor imaging, myelin water imaging, magnetization transfer imaging, and other MRI-based quantitative measures may provide objective evidence of structural brain maturation beyond conventional developmental assessments. Separate evaluation of these studies is expected to improve understanding of the biological effects of iron supplementation on early brain myelination and to guide future research using standardized imaging outcomes (5, 6).
